# Regeneration of Pulmonary Tissue in a Calf Model of Fibrinonecrotic Bronchopneumonia Induced by Experimental Infection with *Chlamydia psittaci*

**DOI:** 10.3390/ijms21082817

**Published:** 2020-04-17

**Authors:** Elisabeth M. Liebler-Tenorio, Jacqueline Lambertz, Carola Ostermann, Konrad Sachse, Petra Reinhold

**Affiliations:** 1 Institute for Molecular Pathogenesis, Friedrich-Loeffler-Institut, Federal Research Institute for Animal Health, Naumburgerstr. 96a, 07743 Jena, Germany; Jacqueline.Lambertz@cvua-rrw.de (J.L.); Carola.Heike.Ostermann@googlemail.com (C.O.); konrad.sachse@gmx.net (K.S.); Petra.Reinhold@fli.de (P.R.); 2 Chemisches und Veterinäruntersuchungsamt Rhein-Ruhr-Wupper (CVUA-RRW), Deutscher Ring 100, 47798 Krefeld, Germany; 3 Institute of Bioinformatics, Friedrich-Schiller-Universität Jena, Leutragraben 1, 07743 Jena, Germany

**Keywords:** lung, pneumonia, necrotizing, regeneration, model, bovine, chlamydia

## Abstract

Pneumonia is a cause of high morbidity and mortality in humans. Animal models are indispensable to investigate the complex cellular interactions during lung injury and repair in vivo. The time sequence of lesion development and regeneration is described after endobronchial inoculation of calves with *Chlamydia psittaci.* Calves were necropsied 2–37 days after inoculation (dpi). Lesions and presence of *Chlamydia psittaci* were investigated using histology and immunohistochemistry. Calves developed bronchopneumonia at the sites of inoculation. Initially, *Chlamydia psittaci* replicated in type 1 alveolar epithelial cells followed by an influx of neutrophils, vascular leakage, fibrinous exudation, thrombosis and lobular pulmonary necrosis. Lesions were most extensive at 4 dpi. Beginning at 7 dpi, the number of chlamydial inclusions declined and proliferation of cuboidal alveolar epithelial cells and sprouting of capillaries were seen at the periphery of necrotic tissue. At 14 dpi, most of the necrosis had been replaced with alveoli lined with cuboidal epithelial cells resembling type 2 alveolar epithelial cells and mild fibrosis, and hyperplasia of organized lymphoid tissue were observed. At 37 dpi, regeneration of pulmonary tissue was nearly complete and only small foci of remodeling remained. The well-defined time course of development and regeneration of necrotizing pneumonia allows correlation of morphological findings with clinical data or treatment regimen.

## 1. Introduction

Pneumonia affects large proportions of the human population worldwide [1]. Community acquired pneumonia (CAP), which is defined as infection not acquired during hospitalization or ventilation, is the most common type of pneumonia and the most frequent cause of morbidity and mortality by infection in developed countries [2]. The annual incidence in adults is 1.5–1.7 per 1000 in Europe [3]. CAP can be caused by viral and bacterial pathogens. Infections with *Streptococcus pneumoniae* and *Haemophilus influenzae* are especially frequent [2,4], but a variety of other bacteria including the intracellular pathogens, *Mycoplasma pneumoniae, Chlamydia (C.) pneumoniae, C. psittaci, Coxiella burnetii* and *Legionella pneumophila* can also be involved [3]. Studies focusing on *Chlamydia* and *Mycoplasma* in patients with CAP indicate that these infections are more frequent than commonly reported, especially in children [5,6,7]. Infections with *C. psittaci* are about twice as frequent as with *C. pneumoniae* [6].

Infection models involving these pathogens are relevant to elucidate host reactions to the pathogen, development and resolution of tissue lesions and to evaluate treatment options. Only animal models can truly reflect the complex cellular interactions during lung injury and repair [8,9]. The search for appropriate animal models of respiratory disease in humans has a long history and is still ongoing [10]. Criteria to optimize experimental research were redefined recently [11]. While mouse models are most frequently used for being cost and time efficient and offering many options for genetic tracing and immunological monitoring, models in domestic animal species, as presented here, possess their own specific advantages, i.e., lung physiology and structure resembling more closely that of humans and spontaneous disease and lesions being comparable to those occurring in humans [8,11,12].

Symptoms of pneumonia are the result of pulmonary alveoli filling with exudate and thus preventing gas exchange. One of the complications of bacterial CAP is necrotizing pneumonia [13,14]. In humans, necrotizing pneumonia is most commonly seen in *Staphylococcus aureus*, *Streptococcus pneumoniae* or *Klebsiella pneumoniae* infections and may occur in both children and adults [13,15]. Conservative versus surgical treatment is subject to controversial debate [13,16,17]. While long-term effects with higher rates of mortality have been reported in elderly patients [18], full resolution within a few months occurred in most children [16].

In cattle and pigs, necrotizing lesions are observed in a number of bacterial pneumonias. The progression to necrotizing lesions may be due to virulence factors of microorganisms or to host reactions, which are more exudative especially in cattle [13]. Experimental infection of calves with *C. psittaci* was reported to progress to necrotizing lesions, with clinical course, pulmonary dysfunction and systemic host reactions having been well characterized [19,20,21,22]. In contrast to the general consensus that the outcome of pulmonary necrosis consists of fibrotic scars, sequestra or abscesses, complete healing was observed. In the following, the time sequence from tissue injury to regeneration is described based on qualitative histological data with the aim of providing a fundamental characterization of a necrotizing pneumonia model.

## 2. Results

### 2.1. Clinical Signs, Acute Phase Response and Pulmonary Dysfunctions

Details of clinical signs and pulmonary dysfunctions induced by the pathogen were reported elsewhere [19,20,21,22]. In brief, intrabronchial inoculation of 10^8^ inclusion forming units (ifu) of *C. psittaci* per calf resulted in acute respiratory illness characterized by fever, dyspnea, dry cough, hyperemic conjunctivae and enlarged mandibular lymph nodes. Respiratory signs were accompanied by signs of a systemic inflammatory response, i.e., elevated heart rates (mild tachycardia), reduced appetite and dullness.

During the period of acute illness (i.e., 2–4 days post inoculation, dpi), blood gas analysis revealed hypoxemia. Pulmonary function testing indicated both obstructive and restrictive pulmonary disorders. The resulting pattern of spontaneous breathing was characterized by a reduction of tidal volume by about 25%, a doubling of respiratory rate, and consequently by a significant increase of minute ventilation to about 150%. Although acute clinical signs decreased and general health improved rapidly from 5 dpi onwards, alterations in respiratory mechanics, acute phase reaction (decreased blood concentrations of albumin and elevated blood concentration of lipopolysaccharide binding protein) and disorders in acid-base equilibrium lasted until 10–11 dpi. By the end of this study (37 dpi), the remaining three calves appeared clinically inconspicuous.

Data of rectal temperature, respiratory rate and tidal volume are given in Table S1 in absolute numbers, while relative changes of these parameters are included in Table 1.

### 2.2. Pulmonary Lesions

#### 2.2.1. At 2 dpi

**Figure 1:**
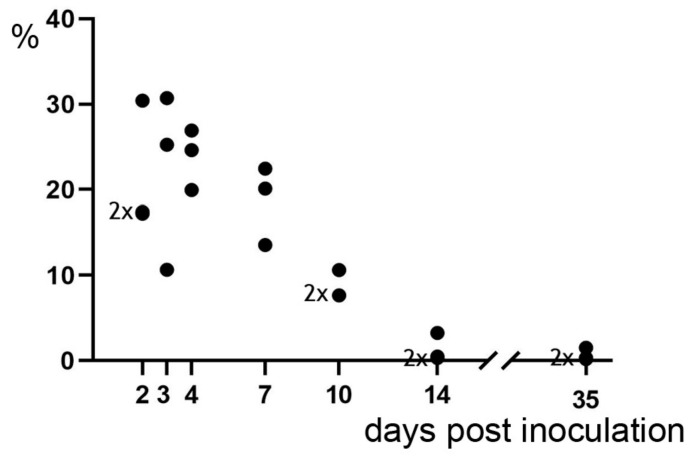
Percentage of pulmonary tissue with lesions at 2, 3, 4, 7, 10, 14 and 35/37 dpi. Each point in the diagram represents one or two (2×) individual calves.

**Figure 2:**
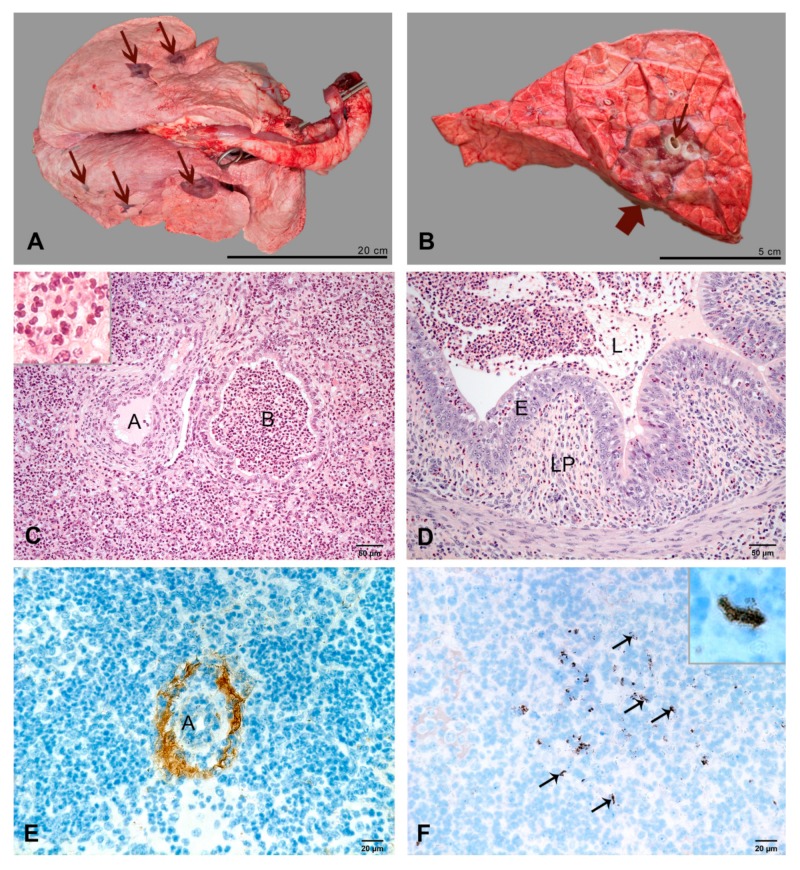
Pulmonary lesions at 2 dpi. (**A**) Macroscopic appearance and distribution of pulmonary lesions (dark red, arrows). (**B**) Section through the left basal lobe. A circumscribed, dark red and sunken lesion (thick arrow) is centered around a bronchus (thin arrow). (**C**) A bronchiolus (B) and the surrounding alveoli are distended by neutrophils. The wall of the arteriole (A) is infiltrated with neutrophils. Inset: higher magnification of neutrophils in an alveolus. HE-stain. (**D**) Numerous neutrophils in the lamina propria (LP), epithelium (E) and lumen (L) of a bronchus. HE-stain. (**E**). Fibrin precipitates (brown) surrounding a small arteriole (A). IHC, factor VIII. (**F**) Few chlamydial inclusions in type 1 alveolar epithelial cells (arrows, examples). Inset: higher magnification of a chlamydial inclusion. IHC, chlamydia.

In two calves 17% of pulmonary tissue and in one calf 30% of pulmonary tissue were dark red, firm with a wet cut surface and pus draining from airways indicating purulent bronchopneumonia (Figure 1 and Figure 2A). Lesions were located around bronchioles at the inoculation sites and often had cylindrical shape (Figure 2B). They were associated with mild fibrinous pleuritis in two calves.

By histology, lesions had a lobular distribution with numerous neutrophils and protein-rich exudate in alveoli, bronchioli and bronchi (Figure 2C,D). The walls and perivascular spaces of small arterioles at the periphery of lesions were thickened by protein-rich edema, fibrin precipitates and neutrophils (Figure 2C,E). Fibrin thrombi obstructed blood vessels. There were areas of necrosis in the vascular walls. Lymphatics in subpleural and interlobular connective tissue contained fibrin thrombi and a few neutrophils. Low numbers of chlamydial inclusions were found in type 1 alveolar epithelial cells (AEC1) and occasionally in neutrophils and macrophages in the exudate (Figure 2F).

#### 2.2.2. At 3 dpi

The extent of pulmonary lesions varied from 11% to 31% (Figure 1). Lesions were firm, grey-red marbled, had a dry cut-surface and were associated with fibrinous pleuritis.

**Figure 3:**
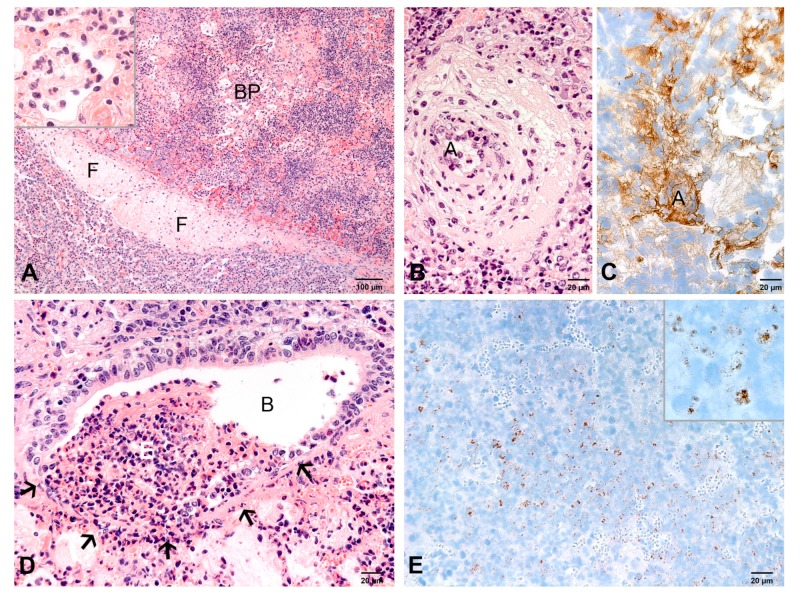
Pulmonary lesions at 3 dpi. (**A**) Lobular fibrinopurulent bronchopneumonia (BP) and fibrin thrombi (F) in interlobular lymphatics. Inset: higher magnification of an alveolus filled with fibrin and neutrophils. HE-stain. (**B**) Severe exudation throughout the wall and in the perivascular space of an arteriole (A). HE-stain. (**C**) Diffuse precipitates of fibrin (brown) in the wall and perivascular space of an arteriole (A) extending into the alveoli. IHC, factor VIII. (**D**) Necrosis (arrows) in the wall of a bronchiolus (B). The lumen is filled with exudate (E). HE-stain. (**E**) Numerous small chlamydial inclusions (brown) in neutrophils and macrophages. Inset: higher magnification of multiple chlamydial inclusions. IHC, chlamydia.

By histology, the fibrinopurulent pneumonia and severe vasculopathy had a lobar distribution and were more extensive than at 2 dpi (Figure 3A,B). Foci of necrosis were more frequent in bronchiolar epithelium and vascular walls (Figure 3B,C). There were small areas of complete necrosis of pulmonary tissue. In these areas, diffuse labeling of fibrin using immunohistochemistry for factor VIII was seen (Figure 3C), but no epithelial cells were labeled for cytokeratin.

Lesions in interlobular septa and pleura had increased in severity. Chlamydial inclusions were numerous in neutrophils and macrophages (Figure 3E), but rare in alveolar epithelial cells.

#### 2.2.3. At 4 dpi

**Figure 4:**
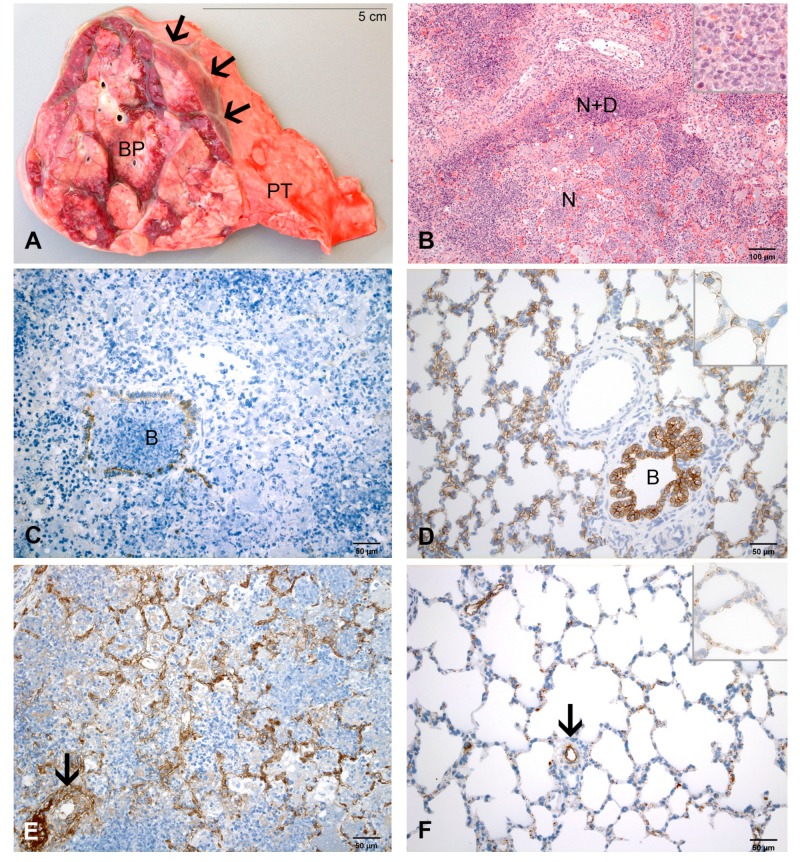
Pulmonary lesions at 4 dpi. (**A**) Section through a basal lobe. The lobular lesion (BP) bulges above the surface of the surrounding pulmonary tissue (PT). The cut surface is drey and marbled grey-red. Interlobular septae and subpleural space are dilated by fibrinous exudate (arrows). (**B**) A large area of necrosis (N) is demarcated by neutrophils and cellular detritus (N+D). Inset: higher magnification of necrosis. HE-stain. (**C**) Epithelial cells are labelled only in segments of the bronchiolar wall (B). There is no labelling of epithelial cells in the surrounding alveoli. IHC, cytokeratin. (**D**) Labelling of epithelial cells in a bronchiolus (B) and surrounding alveoli in the healthy lung of a control calf. Inset: higher magnification of AEC1 in alveolar septae. IHC, cytokeratin. (**E**) Accumulation of fibrin in the wall and perivascular space of an arteriole (arrow). Interalveolar capillaries are dilated by fibrin thrombi (brown). IHC, factor VIII. (**F**) Labeling of factor VIII (brown) is restricted to endothelial cells (arteriole as example, arrow) and thrombocytes in the healthy lung of a control calf. Inset: higher magnification capillaries in alveolar septae. IHC, factor VIII.

Pulmonary lesions varied between 20% and 27% (Figure 1). They were firm, grey-red marbled, bulging and associated with fibrinous pleuritis (Figure 4A).

By histology, lesions in airways, blood vessels and pleura were as described at 3 dpi. Areas of necrosis had expanded (Figure 4B). The inflammatory exudate consisted of neutrophils, fibrin and cellular detritus (Figure 4B). There was a more extensive loss of alveolar epithelial cells than at 3 dpi (Figure 4C,D). Labeling of factor VIII revealed massive accumulations of fibrin in the walls and around arterioles and in the capillaries of interalveolar septa (Figure 4E,F). Several bronchioles and blood vessels were completely obstructed by fibrin. Chlamydial inclusions were numerous in neutrophils, macrophages and detritus in areas of necrosis.

#### 2.2.4. At 7 dpi

Gross pulmonary lesions were as described at 4 dpi, but only 14–22% of the pulmonary volume was affected (Figure 1). Pleural lesions had progressed from fibrinous to fibroblastic.

**Figure 5:**
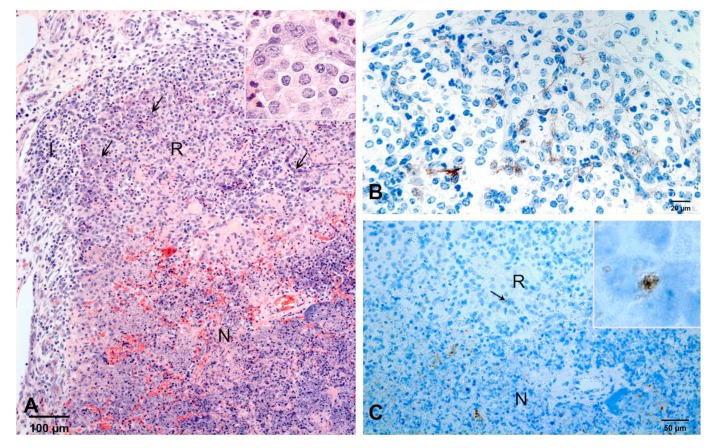
Pulmonary lesions at 7 dpi. (**A**) A few, small groups of epithelial cells (arrows, examples) form a small zone of regeneration (R) at the periphery of a necrosis (N). There is an infiltrate (I) of macrophages, lymphocytes and plasma cells in the adjacent interlobular septum. Inset: higher magnification of a rudimentary alveolar wall. HE-stain. (**B**) Higher magnification of the zone of regeneration. A few cells are weakly positive for cytokeratin (brown). IHC, cytokeratin. (**C**) Chlamydial inclusions (brown) predominate in the necrosis (N) and are rare (arrow) in the zone of regeneration (R). Inset: higher magnification of a chlamydial inclusion in the zoe of regeneration. IHC, chlamydia.

By histology, lesions were characterized by multiple confluent areas of necrosis (Figure 5A). A few weakly cytokeratin-positive cells were present multifocally at the periphery of the necrotic tissue (Figure 5A,B). These epithelial cells were single or formed small aggregates. Airways were filled with macrophages and neutrophils. Compared to 4 dpi, the number of neutrophils was lower and the number of macrophages increased. Moderate infiltrates of lymphocytes and plasma cells were present around bronchi, bronchioles, altered arterioles and in pulmonary tissue adjacent to interlobular septa (Figure 5A). Fibroblasts and delicate collagen fibers had formed in the thickened interlobular septa and subpleural space. Chlamydial inclusions were predominantly present in areas of necrosis (Figure 5C).

#### 2.2.5. At 10 dpi

**Figure 6:**
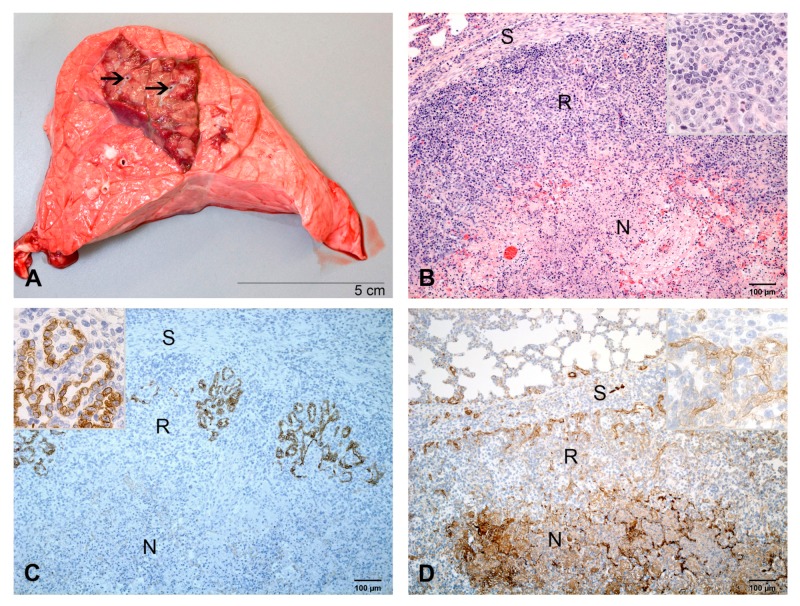
Pulmonary lesions at 10 dpi. (**A**) Section through a basal lobe. The greyish lesion centered round a bronchus (arrow) is demarcated by hyperemia. (**B**) A broad zone of regeneration (R) with multifocal epithelial proliferates surrounds the necrosis (N). Infiltrates of macrophages, lymphocytes and plasma cells are especially prominent next to the interlobular septum (S). Inset: higher magnification of the zone of regeneration. HE-stain. (**C**) Multiple foci of epithelial proliferates (brown) in the zone of regeneration (R) form alveolar structures lined with cuboidal cells. Inset: higher magnification cuboidal epithelial cells. IHC, cytokeratin. (**D**) Capillaries (brown) originating from the interlobular septum (S) pass into the zone of regeneration (R). Fibrinous exudate (brown) is present within the necrosis (N). Inset: higher magnification of capillaries. IHC, factor VIII.

Pulmonary lesions were grey, demarcated by hyperemia and firm (Figure 6A). They amounted to 8% in two calves and 11% of pulmonary volume in one calf (Figure 1) Pleural lesions were fibroblastic.

By histology, wide zones of regenerating pulmonary tissue were surrounding areas of necrosis (Figure 6B). The zone of regeneration was characterized by multifocal epithelial cell aggregates forming irregular tubular and alveolar structures (Figure 6C). Epithelial cells had the cuboidal shape of AEC2 and mitotic figures were frequent (Figure 6B inset). Factor VIII-positive endothelial shoots originated from interlobular septa and passed into the zone of regeneration (Figure 6D). Infiltrates of macrophages, lymphocytes and plasma cells were present throughout the zone of regeneration, especially along interlobular septa, and around arterioles, bronchioles and bronchioli (Figure 6A). They formed multifocal lymphoid aggregates. The exudate in bronchioli and bronchi was lined by epithelial cells resulting in bronchiolitis obliterans. A few fibroblasts and delicate collagen fibers were observed in the bronchiolitis obliterans, perivascular space and interalveolar septa. The distribution of chlamydial inclusions was as described at 7 dpi, but their number was lower.

#### 2.2.6. At 14 dpi

**Figure 7:**
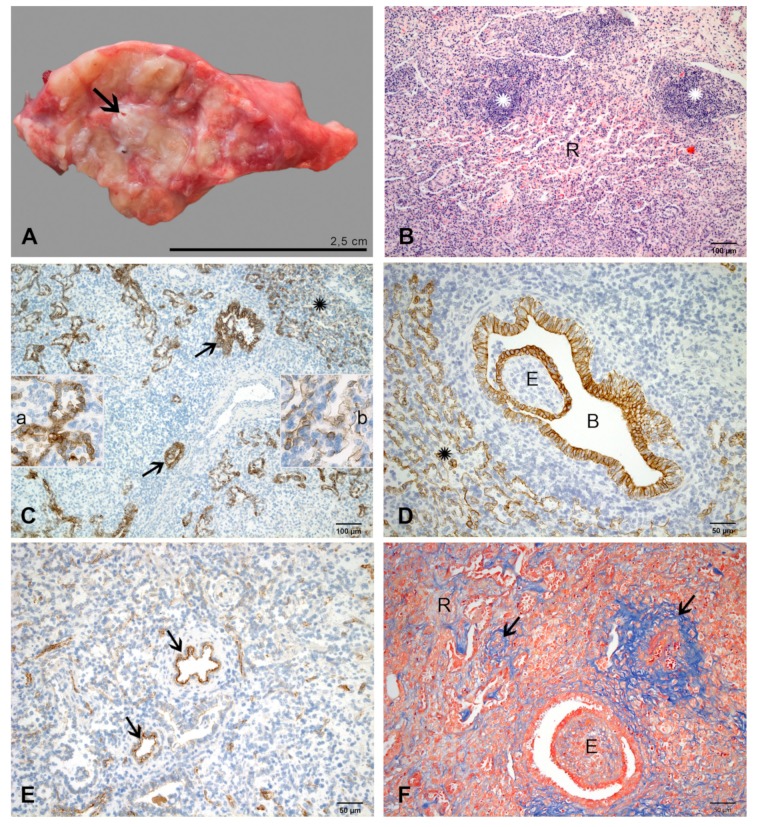
Pulmonary lesions at 14 dpi. (**A**) Section through a middle lobe. The small lesions (white) are centered around a bronchus (arrow) and not well demarcated. (**B**) Extensive regeneration (R) of pulmonary tissue with thick interalveolar septa and narrow alveolar spaces; multifocal organized lymphoid tissue (stars) in the interlobular septum. HE-stain. (**C**) Cuboidal epithelial cells (brown) form irregular alveolar spaces; an area with more progressed regeneration is indicated (star). Bronchiolitis obliterans is seen in most airways (arrows). Inset a: higher magnification of cuboidal epithelial cells forming alveolar spaces. Inset b: higher magnification of delicate, elongated epithelial cells in the area with progressed regeneration. IHC, cytokeratin. (**D**) Higher magnification of more regular alveolar structures lined by type 2 alveolar epithelial cells (star) and the exudate (E) within a bronchiolus (B) lined by cuboidal epithelial cells. IHC, cytokeratin. (**E**) Factor VIII labels endothelial cells in blood vessels (arrows, examples). There is no exudation of fibrin into and through vascular walls. IHC, factor VIII. (**F**) Fibrinous exudate within and around arteriolar walls (arrows) is replaced by collagen fibers (blue). Increased collagen is present in interalveolar septa in the area of regeneration (R) and in the exudate (E) organized by bronchiolitis obliterans. Azan stain.

Pulmonary lesions were reduced to 3% of pulmonary volume in one and less than 1% in two calves (Figure 1). They were firm, white, poorly demarcated (Figure 7A) and frequently accompanied by fibrous adhesions between pulmonary lobes as well as between pulmonary lobes and thoracic wall.

By histology, lesions were characterized by extensive regeneration of pulmonary tissue and only small foci of necrosis (Figure 7B). In areas of regeneration, cuboidal epithelial cells formed irregular alveolar spaces (Figure 7C). Lesions in airways were organized by bronchiolitis and bronchitis obliterans (Figure 7D). Alveolar spaces were separated by thick interalveolar tissue, which contained capillaries originating from subpleural tissue and interlobular septa, and diffuse infiltrates of macrophages, lymphocytes and plasma cells (Figure 7A,B,D). Organized lymphoid tissue forming tertiary lymphoid tissue in interlobular septa and bronchus-associated lymphoid tissue (BALT) around airways and blood vessels was more abundant than at 10 dpi (Figure 7B). There was no leakage of fibrin through arteriolar walls (Figure 7E). The fibrinous exudate in the wall and around arterioles was replaced by several layers of collagen fibres, which compressed the vascular lumen (Figure 7E,F). An increased amount of collagen fibers was also observed in interalveolar and interlobular septa, and in the bronchiolitis obliterans. Chlamydial inclusions were seen in very few alveolar macrophages.

#### 2.2.7. At 35/37 dpi

**Figure 8:**
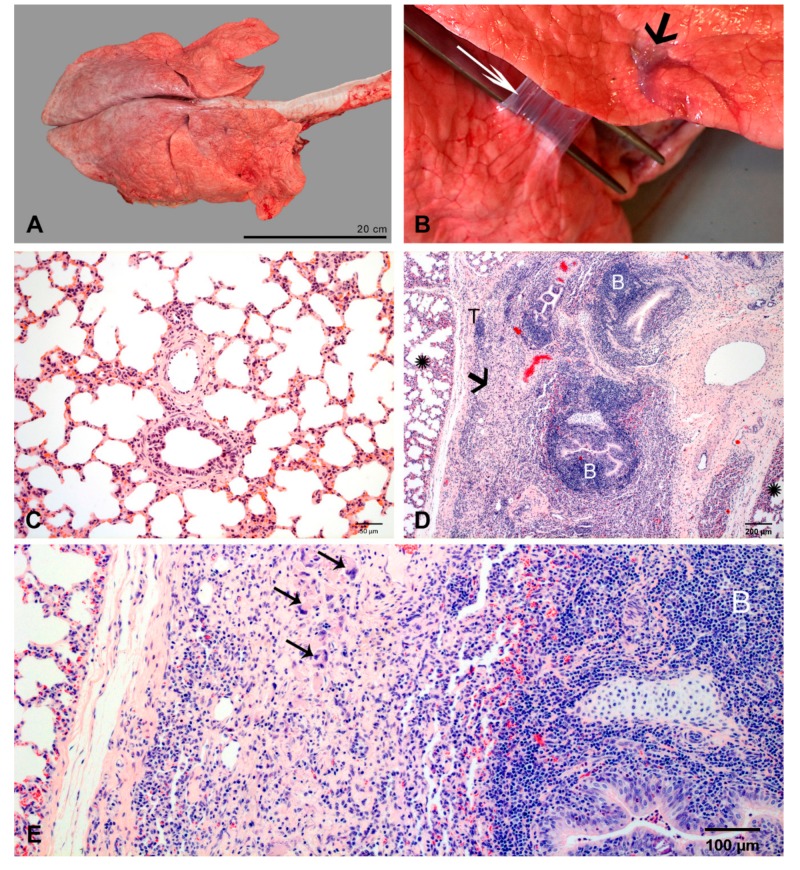
Pulmonary lesions at 35/37 dpi. (**A**) Gross appearance of lung after regeneration. (**B**) Very small lesion in a middle lobe (short arrow) and focal fibrous pleuritis (long arrow) between middle lobe (M) and basal lobe (B). (**C**) Normal pulmonary morphology. HE-stain. (**D**) Small lesion surrounded by normal lung tissue (stars); lesions are characterized by infiltrates of macrophages and multinucleated giant cells (arrow), hyperplastic BALT (B, examples) and tertiary lymphoid tissue (T). HE-stain. (**E**). Higher magnification of the area indicated by the arrow in Figure 8D: infiltrates of macrophages and multinucleated giant cells (arrows, examples), and hyperplastic BALT (B). HE-stain.

Lungs had a normal appearance (Figure 8A). Only a few, very small foci of soft, sunken, dark red pulmonary tissue (2% of total pulmonary volume in one calf and less than 1% in two calves) associated with mild focal fibrous pleuritis were found (Figure 1 and Figure 8B).

By histology, airways, blood vessels and alveolar compartment had completely normal morphology in most of the pulmonary tissue (Figure 8C). BALT and diffuse immune cells had regressed to normal amounts. There were no tertiary lymphoid tissue and no signs of fibrosis.

The small lesions were characterized by infiltrates of macrophages and multinucleated giant cells embedded in vascularized fibrotic tissue (Figure 8D,E). Lymphocytic infiltrates were organized as peribronchial and perivascular BALT, and tertiary lymphoid tissue in the interlobular septa. Chlamydial inclusions were not detected.

## 3. Discussion

A bovine model of respiratory infection with the zoonotic pathogen *C. psittaci* was previously established [19]. This model has been used to investigate the course of disease, effects on pulmonary functions as well as shedding, transmission and zoonotic potential of *C. psittaci* [20,21,22]. The infection model was very efficient, since all calves inoculated with the pathogen developed pneumonia, but none of the controls. The model was highly reproducible, and therefore repeatedly used to examine outcomes of different treatment regimens [23,24]. The localization of lesions corresponded to the inoculation sites and was distinct from field infections that have a cranioventral distribution [25]. Thus, the model consistently allows induction of lesions at predetermined sites of the lung.

For this qualitative histological study, groups of 3 of the 21 calves inoculated were necropsied at predetermined days post inoculation. Although the number of animals is low, the validity of observations is supported by the concordance of histological findings and consistent progression patterns. The morphological evaluation of the lung at sequential time points after inoculation revealed the potential of this model to study tissue reactions from initial stages of inflammation to extensive necrosis, up to stepwise organization and regeneration. The reactions observed are matching well with the steps reported as general reactions of pulmonary tissue to an insult [26]: primary lesions are often amplified through inflammatory host reactions. If the host is capable of limiting damage and proliferation of the infectious agent, organization begins. This may result in reparation, resolution or remodeling. Reparation implies repopulation with epithelial cells, but dysfunctional tissue structure. In a remodeled lung, pulmonary tissue is replaced with connective tissue, which is dysfunctional and may cause ectasia and emphysema of adjacent tissue. A successful return to normal structure and function is termed resolution or regeneration.

In the model presented, the initial insult was the intrabronchial inoculation with *C. psittaci,* which has a tropism for epithelial cells [27]. This resulted in a limited infection of AEC1. Infected alveolar epithelial cells have the potential to produce and release cytokines, e.g., the proinflammatory cytokine IL8, which is highly chemotactic for neutrophils, chemokines and growth factors initiating the augmentation [28,29]. Chlamydiae are released during the replication cycle from AEC1 into the alveolar lumen where they get into contact with alveolar macrophages. Bacterial LPS may induce necrosis of alveolar macrophages and release of IL-1α, which lead to a loss of vascular integrity and thus increase the influx of neutrophils from the pulmonary vasculature into the alveoli and airways [30]. Neutrophils display unique migration mechanisms in the lung resulting in particularly high numbers [31]. Inflammatory mediators enhance neutrophil activity and their deleterious effect on endothelium and epithelium [32,33]. The severe exudation and the necrosis of bronchiolar epithelium observed in the calves are most likely due to host reactions and not an effect of chlamydial replication, since no chlamydial inclusions were present in vascular endothelium or airway epithelium.

The massive release of protein-rich exudate and fibrin as seen at 3 dpi and 4 dpi is a common reaction in cattle and has been attributed to an imbalance of pro- and antifibrinolytic factors [34]. Fibrin can further increase vascular permeability, influence the expression of inflammatory mediators and alter migration and proliferation of various cell types [35]. Fibrin thrombi may occlude capillaries and arterioles and thus reduce perfusion; fibrinous exudate within alveoli prevents ventilation. This results in tissue hypoxia and, eventually, necrosis as observed as fibrinonecrotic bronchopneumonia with highest severity at 4 dpi.

While the number of neutrophils continuously declined, the number of macrophages increased during this initial phase. Airway macrophages possess high phagocytic activity to remove debris and exudate, which also included chlamydiae. Processing and presentation of chlamydiae and chlamydial antigens by macrophages may enhance the pathogen-specific immune response. Macrophages also play an important role in the downregulation of immune response and in tissue repair [30,36]. Sloughed epithelial cells, dying neutrophils and microvesicles as encountered in areas of necrosis comprise a rich depot of phosphatidyl serines that can reprogram macrophages from a proinflammatory to an antiinflammatory and prorepair state [9].

In patients with localized destructive processes such as necrotizing pneumonia, which may progress to cavitating lesions, it has been postulated that there is no return to preexisting tissue architecture [37]. In our model, tissue necrosis but no cavitation occurred. Under field conditions, fibrinonecrotic bronchopneumonia in domestic animals rarely resolves completely and sequelae, like sequestra, abscesses, gangrene, fibrosis, scars or chronic pleuritis, are frequent [38]. This may be due to the ability or failure of the host to clear the pathogen and terminate chronic inflammation. After the initial infection of AEC1, chlamydial inclusions were present in continuously decreasing numbers in neutrophils and macrophages. At 14 dpi, they had been almost cleared. From 7 dpi to 14 dpi, the infiltration of lymphocytes representing an adaptive immune response continuously increased [39]. Besides diffuse lymphoid infiltrates, organized structures developed as tertiary lymphoid tissue in the interalveolar septa and as BALT around airways. This local immune reaction was transient and had disappeared at 35/37 dpi.

At the same time, small aggregates of epithelial cells, as first signs of tissue repair, were observed at the edge of necrosis. The pulmonary epithelium does not exhibit a constant turnover, but can respond robustly after injury to replace damaged cells. In the alveolar niche, especially type 2 alveolar epithelial cells (AEC2) have an enormous reparative potential [40,41,42]. They clonally generate more AEC2 [43]. The cuboidal AEC2 grow, proliferate, follow the basement membrane and secrete basement membrane components, but are inefficient for gas exchange [44].

The multifocal distribution of epithelial cell regenerates at the edge necrosis may be attributable to the fact, that a few AEC2 were retained at these sites and served as starting points [43]. This process may have been supported by remnants of basal membrane, an important scaffold for AEC2 [45]. Recently it was shown that even functionally mature AEC1 could replicate and generate AEC2 [46]. Thus, the delicate epithelial cells observed at 7 dpi might also represent surviving AEC1. Cuboidal epithelial cells interpreted as AEC2 increasingly replaced areas of necrosis. The process started at the periphery and moved towards the center of necrotic tissue leaving only a few small foci of necrosis at 14 dpi. Efficient epithelialization is likely to be crucial for the prevention of pathologic lung remodeling [26].

The cuboidal epithelial cells formed organized alveolar spaces. Maintenance of alveolar units requires complex interactions between various cell types including epithelial cells, endothelial cells, mesenchymal cells, macrophages and other immune cells, and a great variety of mediators [9,47]. Vascularization in areas of re-epithelialization can originate from multipotent mesenchymal stem cells or from blood vessels in adjacent tissue by sprouting. Capillary sprouts originating from the connective tissue of interlobular septa adjacent to areas of necrosis were seen as early as 10 dpi. The sprouting process involves several specialized types of endothelial cells. Vascular endothelial growth factor or inflammatory cytokines induce the formation of tip cells that use filopodia to sense environmental cues, translate these into dynamic processes and express matrix metalloproteinases for invasion [48]. Tip cells use extracellular matrix as a scaffold, provided in this case by the epithelial cells and basement membrane. Stalk cells elongate the sprouting vessels, form the lumen and connect it to the circulation, while phalanx cells stabilize the new vessels and optimize their function [48]. The combination of vascularization and epithelialization contributed to the successful neo-alveolarization observed. The final step in this process is the maturation of cuboidal alveolar epithelial cells to AEC1 and thus functional alveoli [8,40]. This took place between 14 dpi and 37 dpi when the last calves were examined.

The fibrinous exudate and growth factors, e.g., transforming growth factor β, released from inflammatory cells, such as macrophages, and endothelial cells induced immigration of fibroblasts and production of collagen and extracellular matrix. The first fibroblasts and collagen fibers were observed at 7 dpi and distinct fibrosis around arterioles and in the interalveolar septa at 14 dpi. Since there was no permanent or progressive fibrosis in our model, it does not reflect pulmonary fibrosis in humans [37,49], but it is an example that the development of fibrosis can be prevented. The time course of development and resolution of fibrosis in the calves was comparable to that in bleomycin-induced lung fibrosis in mice, where the maximal extent of fibrosis was found at 14 days after a single instillation and subsided after treatment was withdrawn [50].

There was a good correlation between clinical data, deterioration of lung function and pulmonary lesions (Table 1). The more extensive lesions during the initiation phase were associated with systemic signs, e.g., fever, increased respiratory rate and decreased tidal volume. As soon as the volume of pulmonary lesions decreased and organization started, lung function and clinical signs returned to normal values. This occurred before regeneration was complete, because cattle ventilate only about 30% of the total lung volume when breathing spontaneously under resting conditions [51].

In conclusion, the experimental infection of calves with *C. psittaci* allows (1) to dissect the tissue processes involved in the development and resolution of inflammation, lesion development, neo-alveolarization, revascularization, waxing and waning of immune cell infiltrates, and fibrosis in the lung, (2) to correlate clinical with morphological findings and (3) to investigate the influence of treatment regimens on lung regeneration. These qualitative histological data may serve as basis for further in-depth studies using more advanced methods.

## 4. Materials and Methods

### 4.1. Animals

Forty-two conventionally raised calves (Holstein-Friesian, male) were included in this study. Animals originated from a farm without history of *Chlamydia*-associated health problems. Before the study, the herd of origin was regularly tested for the presence of Chlamydiae by the National Reference Laboratory for Psittacosis. Calves were purchased at the age of 14–28 days weighing between 42.2 and 71.2 kg. Animals were included in the study after a quarantine period of at least 20 days and confirmation of a clinically healthy status.

Throughout the entire study, animals were reared under standardized conditions (room climate: 18–20 °C) and in accordance with international guidelines for animal welfare. Nutrition included commercial milk replacer and coarse meal. Water and hay were supplied ad libitum. None of the given feed contained antibiotics.

This study was carried out in strict accordance with European and National Law for the Care and Use of Animals. The protocol was approved by the Committee on the Ethics of Animal Experiments and the Protection of Animals of the State of Thuringia, Germany (Permit Number: 04-002/07, 18 December 2007). All experiments were done in a containment of biosafety level 2 under supervision of the authorized institutional Agent for Animal Protection. Bronchoscopy to inoculate the pathogen was strictly performed under general anesthesia. During the entire study, every effort was made to minimize suffering.

### 4.2. Experimental Design

Twenty-one calves were inoculated with *C. psittaci* and twenty-one calves served as controls. The inoculum was placed at eight defined pulmonary sites by bronchoscope. Each calf underwent daily clinical examination. Groups of three calves were euthanized at 2, 3, 4, 7, 10, 14 and 35 or 37 dpi. At necropsy, distribution and extent of pulmonary lesions were determined and samples collected for histological, immunohistochemical and microbiological investigations.

### 4.3. Inoculum and Inoculation

*C. psittaci* strain 02DC15 was isolated at the Friedrich-Loeffler-Institut, Jena, Germany, from an aborted calf fetus in 2002. The isolate was classified as *C. psittaci* genotype A-VS1 by DNA microarray testing and *omp*A gene sequencing [52]. Chlamydiae were propagated in buffalo green monkey kidney cell culture using standard procedures [53]. Frozen stocks of strain 02DC15 were diluted to the required titer in stabilizing SPGA medium and used as inoculum in the present trial.

Calves were inoculated with 10^8^ ifu of *C. psittaci* strain 02DC15 in 6 mL stabilizing medium SPGA (containing saccharose, phosphatile substances, glucose and bovine albumin) [54]. Control calves received the same amount of SPGA containing buffalo green monkey kidney cells.

**Figure 9:**
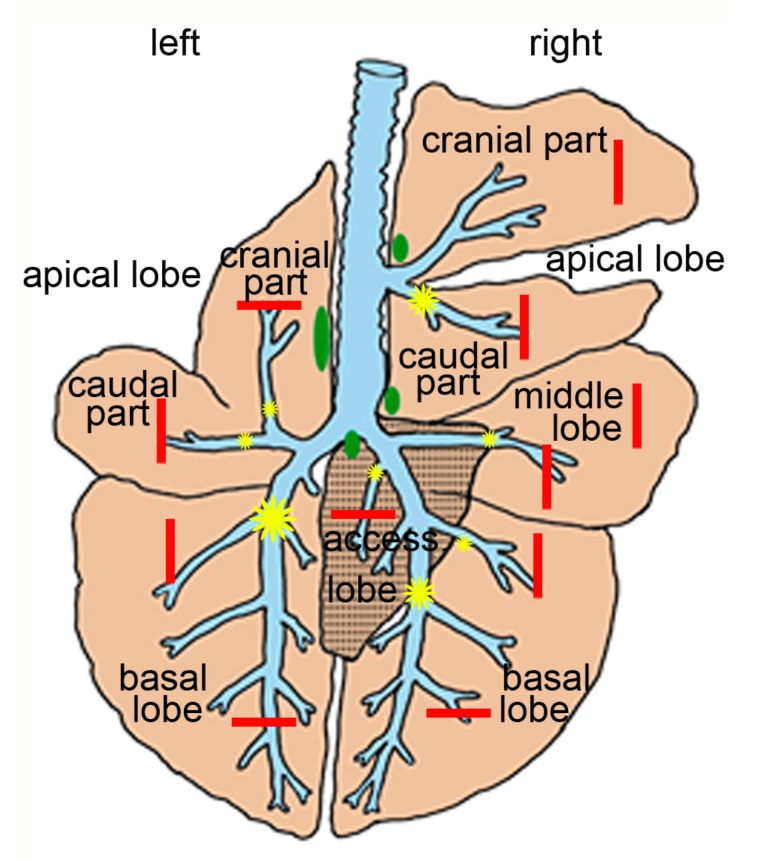
Schematic drawing of bovine lung. Inoculation sites are indicated as yellow stars (small—0.5 mL, middle-sized—1.0 mL and large—1.5 mL), sampling sites as red bars and and regional lymph nodes in green.

The inoculation by bronchoscope has been described in detail [19,55]. In brief, non-fed calves were anesthetized with xylazin (0.2 mg/kg body weight, Rompun 2%, Bayer Vital GmbH, Leverkusen, Germany) and ketamine (1.7 ± 0.3 mg/kg body weight, Ursotamin, Serumwerk Bernburg AG, Bernburg, Germany). A flexible video endoscope (Veterinary Video-Endoscope PV-SG 22–140, Karl Storz GmbH and Co.KG, Germany) was inserted through the oral cavity and 0.5–1.5 mL of inoculum were placed at eight defined pulmonary sites (Figure 9).

### 4.4. Clinical Scoring and Pulmonary Function Testing

Clinical observations were recorded twice daily and included feed intake, rectal temperature, respiratory rate and the presence or absence of clinical signs of diarrhea or respiratory disease. In addition, the appearance of oral mucosa, conjunctivae, skin, hair and dyspnea were assessed daily, and the heart rate was counted. Extremities, umbilicus and mandibular lymph nodes were palpated and inducement of cough was tested. Results were summarized using a 49-point clinical score consisting of subscores for general condition (maximum 8 points), respiratory system (maximum 17 points), cardiovascular system (maximum 13 points) and other organ systems (maximum 11 points) as described [19].

Pulmonary function testing was performed using the impulse oscillometry system (IOS) as described elsewhere [22].

### 4.5. Necropsy, Gross Pathology and Tissue Samples

Always three calves inoculated with chlamydiae and three controls were euthanized at 2, 3, 4, 7, 10, 14 and 35/37 dpi. In deep anesthesia (pentobarbital-sodium, 770 ± 123 mg/10 kg body weight, intravenously, Release, WdT eG, Garbsen, Germany), the trachea was exposed and large clamps were placed distal to the larynx to prevent contamination of the airways by blood or gastric contents. Subsequently, animals were sacrificed by exsanguination. The lung was removed and macroscopic lesions were recorded. To determine the total percentage of pulmonary lesions, the percentage of lesions was subjectively assessed for each lobe and multiplied by the relative percentage of the respective pulmonary lobe of the total lung volume. The volumes had been determined in 18 age-matched calves prior to this study and were measured as displacement of water. For this, the total lung volume was determined; then the different lobes were dissected, the main bronchi closed and their volume measured. On average, the left cranial and caudal apical lobes contributed 5%, the left caudal lobe 34%, the right cranial apical lobe 5%, the right caudal apical lobe 7%, the middle lobe 8%, the right caudal lobe 35% and the accessory lobe 1% to the total lung volume (Figure 9).

Samples were collected from each lung lobe (Figure 9) and fixed in 3.5% neutral buffered formalin. Sites with macroscopic lesions were preferentially sampled. Then a complete necropsy was performed.

### 4.6. Histopathology

Formalin-fixed tissues were embedded in paraffin after 24 h. Formalin-fixed paraffin-embedded (FFPE) tissue sections were stained with hematoxylin and eosin to evaluate lesions. Consecutive sections were stained with azan to demonstrate collagen fibers and by PAS-reaction for glycogen-rich material.

### 4.7. Immunohistochemistry

Consecutive FFPE tissue sections were used to label chlamydiae, epithelial cells and blood vessels by the indirect immunoperoxidase method. As primary antibodies, the anti-chlamydial-LPS antibody ACI-P500 (Progen, Heidelberg, Germany), anti-cytokeratin antibody MNF116 (Dako Denmark, Glostrup, Denmark) and anti-factor VIII polyclonal antiserum (Dako Denmark, Glostrup, Denmark) were used. Peroxidase-labeled sheep anti-mouse IgG (NA 931, GE Healthcare Europe GmbH, Freiburg, Germany) served as secondary antibody for the monoclonal antibodies and peroxidase-labeled goat anti-rabbit IgG for the polyclonal antiserum. Sections were digested with 0.05% proteinase K (Merck, Darmstadt, Germany) for antigen retrieval. Diaminobenzidine was used as chromogen.

### 4.8. Exclusion of Co-Infections

The herd of origin was known to be free of bovine herpes virus 1 and bovine virus diarrhea/mucosal disease virus. Routine microbiological screening revealed that all animals were negative for *Salmonella* infections (fecal swabs) and relevant enteric parasites (fecal smears). To verify relevant respiratory co-pathogens, the presence of *Mycoplasma*, *Pasteurella* or *Mannheimia* spp. was evaluated in nasal swabs taken immediately before challenge and before necropsy as well as in lung tissue samples obtained during necropsy. Neither *Mannheimia haemolytica* nor *Mycoplasma bovis* was detected in any sample. *Pasteurella multocida* and *Mycoplasma bovirhinis* were detected in nasal swabs, but never in any lung tissue sample. Serological findings confirmed that animals did not acquire infections with respiratory viruses relevant in bovines (i.e., bovine respiratory syncytial virus, parainfluenza 3 virus or adenovirus type 3).

## Figures and Tables

**Table 1:** Clinical data, respiratory function, volume and characteristics of pulmonary lesions.

Day of Necropsy in dpi	Absolute Change in Rectal Temperature ^1^ in °C, Median {min; max}	Relative Change in Respiratory Rate ^2^	Relative Change in Tidal Volume per kg b.wW. ^3^	Volume of Pulmonary Lesions	Histologic Characteristics of Pulmonary Lesions
		in %, Median {min; max}			
2 (*n* = 3)	+2.5 {+1.8; +2.6}	+150 {+133; +285}	n.a. ^4^	17 {17; 30}	purulent bronchopneumonia
3 (*n* = 3)	+ 2.4 {+1.9;+2.8}	+200 {+83; +173}	−19 {−38; −3}	25 {11; 31}	fibrinopurulent bronchopneumonia
4 (*n* = 3)	+1.0 {+0.5; +1.5}	+104 {+82; +127}	−27 {−31; −23}	25 {20; 27}	fibrinopurulent to necroticzing bronchopneumonia
7 (*n* = 3)	−0.6 {−0.7; +0.5}	+20 {0; +23}	−2 {−11; −1}	20 {14; 22}	first signs of organization, macrophage infiltration
10 (*n* = 3)	−0.1 {−0.7; +2.0}	0 {−4; 0}	+13 {−3; +19}	8 {8; 11}	cuboidal alveolar epithelial cells, new capillaries
14 (*n* = 3)	+0.1 {−0.1; +0.2}	+23 {+13; +67}	+19 {−1; +40}	0.5 {0.3; 3.3}	progressed organization, lymphocyte infiltration
35/37 (*n* = 3)	-0.1 {−0.4; +0.9}	−22 {−33; −11}	+2 {−2; +6}	0.3 {0.2; 1.5}	regeneration, remodeling (in lesions)

^1^ Absolut change of rectal temperature between the average rectal temperature measured at two different days in the week before challenge (baseline) and the rectal temperature measured at the day of necropsy. ^2^ Respiratory rate was counted in resting animals (in stable). The relative change of respiratory rate was calculated between baseline values (= mean of individual measurements at two different days in the week before challenge) and the respiratory rate counted at the day of necropsy. ^3^ body weight; ^4^ not available (calves necropsied at 2 dpi were too sick to undergo pulmonary function testing).

## Supplementary Materials

Supplementary Materials can be found at https://www.mdpi.com/1422-0067/21/8/2817/s1.

## Abbreviations

AEC1	type 1 alveolar epithelial cells
AEC2	type 2 alveolar epithelial cells
BALT	bronchus-associated lymphoid tissue
C.	chlamydia
CAP	community acquired pneumonia
dpi	days after inoculation
FFPE	formalin-fixed paraffin embedded tissue
ifu	inclusion-forming units
IL	interleukin

## References

[1] Marshall D.C., Goodson R.J., Xu Y., Komorowski M., Shalhoub J., Maruthappu M., Salciccioli J.D. (2018). Trends in mortality from pneumonia in the Europe union: A temporal analysis of the European detailed mortality database between 2001 and 2014. Resp. Res..

[2] Welte T., Torres A., Nathwani D. (2012). Clinical and economic burden of community-acquired pneumonia among adults in Europe. Thorax.

[3] Cillóniz C., Torres A., Niederman M., van der Eerden M., Chalmers J., Welte T., Blasi F. (2016). Community-acquired pneumonia related to intracellular pathogens. Intensive Care Med..

[4] Torres A., Blasi F., Peetermans W.E., Viegi G., Welte T. (2014). The aetiology and antibiotic management of community-acquired pneumonia in adults in Europe: A literature review. Eur. J. Clin. Microbiol. Infect. Dis..

[5] Wubbel L., Muniz L., Ahmed A., Trujillo M., Carubelli C., Mccoig C., Abramo T., Leinonen M., McCracken G.H. (1999). Etiology and treatment of community-acquired pneumonia in ambulatory children. Pediatr. Infect. Dis. J..

[6] Dumke R., Schnee C., Pletz M.W., Rupp J., Jacobs E., Sachse K., Rohde G., CAPNETZ Study Group (2015). Mycoplasma pneumoniae and Chlamydia spp. infection in community-acquired pneumonia, Germany, 2011–2012. Emerg. Infect. Dis..

[7] Spoorenberg S.M.C., Bos W.J.W., van Hannen E.J., Dijkstra F., Heddema E.R., van Velzen-Blad H., Heijligenberg R., Grutters J.C., de Jongh B.M., Ovidius Study Group (2016). *Chlamydia psittaci*: A relevant cause of community-acquired pneumonia in two Dutch hospitals. Neth. J. Med..

[8] Herriges M., Morrisey E.E. (2014). Lung development: Orchestrating the generation and regeneration of a complex organ. Development.

[9] Zemans R.L., Henson P.M., Henson J.E., Janssen W.J. (2015). Conceptual approaches to lung injury and repair. Ann. Am. Thorac. Soc..

[10] Slauson D.O., Hahn F.F. (1980). Criteria for development of animal models of diseases of the respiratory system. Am. J. Pathol..

[11] Bonniaud P., Fabre A., Frossard N., Guignabert C., Inman M., Kuebler W.M., Maes T., Shi W., Stampfli M., Uhlig S. (2018). Optimising experimental research in respiratory diseases: An ERS statement. Eur. Respir. J..

[12] Ireland J.J., Roberts R.M., Palmer G.H., Bauman D.E., Bazer F.W. (2008). A commentary on domestic animals as dual-purpose models that benefit agricultural and biomedical research. J. Anim. Sci..

[13] Tsaia Y.-F., Kub Y.-H. (2012). Necrotizing pneumonia: A rare complication of pneumonia requiring special consideration. Curr. Opin. Pulm. Med..

[14] Krutikov M., Rahman A., Tiberi S. (2019). Necrotizing pneumonia (aetiology, clinical features and management). Curr. Opin. Pulm. Med..

[15] Hilton B., Tavare A.N., Creer D. (2017). Necrotising pneumonia caused by non-PVL Staphylococcus aureus with 2-year follow-up. BMJ Case Rep..

[16] Sawicki G.S., Lu F.L., Valim C., Cleveland R.H., Colin A.A. (2008). Necrotising pneumonia is an increasingly detected complication of pneumonia in children. Eur. Respir. J..

[17] Alifano M., Lorut C., Lefebvre A., Khattar L., Damotte D., Huchon G., Regnard J.-F., Rabbat A. (2011). Necrotizing pneumonia in adults: Multidisciplinary management. Intens. Care Med..

[18] Koivula I., Stén M., Mäkelä P.H. (1999). Prognosis after community-acquired pneumonia in the elderly: A population-based 12-year follow-up study. Arch. Intern. Med..

[19] Reinhold P., Ostermann C., Liebler-Tenorio E., Berndt A., Vogel A., Lambertz J., Rothe M., Rüttger A., Schubert E., Sachse K. (2012). A bovine model of respiratory Chlamydia psittaci infection: Challenge dose titration. PLoS ONE.

[20] Ostermann C., Schroedl W., Schubert E., Sachse K., Reinhold P. (2013). Dose-dependent effects of *Chlamydia psittaci* infection on pulmonary gas exchange, innate immunity and acute-phase reaction in a bovine respiratory model. Vet. J..

[21] Ostermann C., Rüttger A., Schubert E., Schrödl W., Sachse K., Reinhold P. (2013). Infection, disease, and transmission dynamics in calves after experimental and natural challenge with a bovine *Chlamydia psittaci* isolate. PLoS ONE.

[22] Ostermann C., Linde S., Siegling-Vlitakis C., Reinhold P. (2014). Evaluation of pulmonary dysfunctions and acid–base imbalances induced by *Chlamydia psittaci* in a bovine model of respiratory infection. Multidiscip. Respir. Med..

[23] Prohl A., Lohr M., Ostermann C., Liebler-Tenorio E., Berndt A., Schroedl W., Rothe M., Schubert E., Sachse K., Reinhold P. (2015). Enrofloxacin and macrolides alone or in combination with rifampicin as antimicrobial treatment in a bovine model of acute *Chlamydia psittaci* infection. PLoS ONE.

[24] Prohl A., Lohr M., Ostermann C., Liebler-Tenorio E., Berndt A., Schroedl W., Rothe M., Schubert E., Sachse K., Reinhold P. (2015). Evaluation of antimicrobial treatment in a bovine model of acute *Chlamydia psittaci* infection: Tetracycline versus tetracycline plus rifampicin. FEMS Pathog. Dis..

[25] Panciera R.J., Confer A.W. (2010). Pathogenesis and pathology of bovine pneumonia. Vet. Clin. North. Am. Food Anim. Pract..

[26] Beers M.F., Morrisey E.E. (2011). The three R’s of lung health and disease: Repair, remodeling, and regeneration. J. Clin. Investig..

[27] Virok D.P., Nelson D.E., Whitmire W.M., Crane D.D., Goheen M.M., Caldwell H.D. (2005). Chlamydial infection induces pathobiotype-specific protein tyrosine phosphorylation in epithelial cells. Infect. Immun..

[28] Rasmussen S.J., Eckmann L., Quayle A.J., Shen L., Zhang Y.-X., Anderson D.J., Fierer J., Stephens R.S., Kagnoff M.F. (1997). Secretion of proinflammatory cytokines by epithelial cells in response to chlamydia infection suggests a central role for epithelial cells in chlamydial pathogenesis. J. Clin. Investig..

[29] Jahn H.-U., Krüll M., Wuppermann F.N., Klucken A.C., Rosseau S., Seybold J., Hegemann J.H., Jantos C.A., Suttorp N. (2000). Infection and activation of airway epithelial cells by *Chlamydia pneumoniae*. J. Infect. Dis..

[30] Vanella K.M., Wynn T.A. (2017). Mechanism of organ injury and repair by macrophages. Annu. Rev. Physiol..

[31] Kovtun A., Messerer D.A.C., Scharffetter-Kochanek K., Huber-Lang M., Ignatius A. (2018). Neutrophils in tissue trauma of the Skin, bone, and lung: Two sides of the same coin. J. Immunol. Res..

[32] Carden D., Xiao F., Moak C., Willis B.H., Robinson-Jackson S., Alexander S. (1998). Neutrophil elastase promotes lung microvascular injury and proteolysis of endothelial cadherins. Am. J. Physiol..

[33] Ginzberg H.H., Cherapanov V., Dong Q., Cantin A., McCulloch C.A.G., Shannon P.T., Downey G.P. (2001). Neutrophil-mediated epithelial injury during transmigration: Role of elastase. Am. J. Physiol. Gastrointest. Liver Physiol..

[34] Car B.D., Suyemoto M.M., Neilsen N.R., Slauson D.O. (1991). The role of leukocytes in the pathogenesis of fibrin deposition in bovine acute lung injury. Am. J. Pathol..

[35] Wygrecka M., Jablonska E., Guenther A., Preissner K.T., Markart P. (2008). Current view on alveolar coagulation and fibrinolysis in acute inflammatory and chronic interstitial lung diseases. Thromb. Haemost..

[36] Puttur F., Gregory L.G., Lloyd C.M. (2019). Airway macrophages as the guardians of tissue repair in the lung. Immunol. Cell. Biol..

[37] Wallace W.A., Fitch P.M., Simpson A.J., Howie S.E. (2007). Inflammation-associated remodelling and fibrosis in the lung - a process and an end point. Int. J. Exp. Pathol..

[38] Lopez A., McGavin M.D., Zachary J.F. (2007). Respiratory System. Pathologic Basis of Veterinary Disease.

[39] Roan N.R., Starnbach M.N. (2008). Immune-mediated control of chlamydia infection. Cell. Microbiol..

[40] Evans M.J., Cabral L.J., Stephens R.J., Freeman G. (1975). Transformation of alveolar type 2 cells to type 1 cells following exposure to NO_2_. Exp. Mol. Pathol..

[41] Chen F., Fine A. (2016). Stem cells in lung injury and repair. Am. J. Pathol..

[42] Tata P.R., Rajagopal J. (2017). Plasticity in the lung: Making and breaking cell identity. Development.

[43] Barkauskas C.E., Cronce M.J., Rackley C.R., Bowie E.J., Keene D.R., Stripp B.R., Randell S.H., Noble P.W., Hogan B.L. (2013). Type 2 alveolar cells are stem cells in adult lung. J. Clin. Investig..

[44] Fang K.C. (2000). Mesenchymal regulation of alveolar repair in pulmonary fibrosis. Am. J. Respir. Cell. Mol. Biol..

[45] Vracko R. (1974). Basal lamina scaffold-anatomy and significance for maintenance of orderly tissue structure. Am. J. Pathol..

[46] Jain R., Barkauskas C.E., Takeda N., Bowie E.J., Aghajanian H., Wang Q., Padmanabhan A., Manderfield L.J., Gupta M., Li D. (2015). Plasticity of Hopx(+) type I alveolar cells to regenerate type II cells in the lung. Nat. Commun..

[47] Rodríguez-Castillo J.A., Pérez D.B., Ntokou A., Seeger W., Morty R.E., Ahlbrecht K. (2018). Understanding alveolarization to induce lung regeneration. BMC Respir. Res..

[48] De Smet F., Segura I., De Bock K., Hohensinner P.J., Carmeliet P. (2009). Mechanisms of vessel branching: Filopodia on endothelial tip cells lead the way. Arterioscler. Thromb. Vasc. Biol..

[49] Chua F., Gauldie J., Laurent G.J. (2005). Pulmonary fibrosis: Searching for model answers. Am. J. Respir. Cell. Mol. Biol..

[50] Izbicki G., Segel M.J., Christensen T.G., Conner M.W., Breuer R. (2002). Time course of bleomycin-induced lung fibrosis. Int. J. Exp. Pathol..

[51] Veit H.P., Farrell R.L. (1978). The anatomy and physiology of the bovine respiratory system relating to pulmonary disease. Cornell Vet..

[52] Sachse K., Laroucau K., Vorimore F., Magnino S., Feige J., Müller W., Kube S., Hotzel H., Schubert E., Slickers P. (2009). DNA microarray-based genotyping of *Chlamydophila psittaci* strains from culture and clinical samples. Vet. Microbiol..

[53] Goellner S., Schubert E., Liebler-Tenorio E., Hotzel H., Saluz H.P., Sachse K. (2006). Transcriptional response patterns of *Chlamydophila psittaci* in different in vitro models of persistent infection. Infect. Immun..

[54] Bovarick M.R., Miller J.C., Snyder J.C. (1950). The influence of certain salts, amino acids, sugars, and proteins on the stability of rickettsiae. J. Bacteriol..

[55] Prohl A., Ostermann C., Lohr M., Reinhold P. (2014). The bovine lung in biomedical research: Visually guided bronchoscopy, intrabronchial inoculation and in vivo sampling techniques. J. Vis. Exp..

