# Supplementary material for: Regeneration of Pulmonary Tissue in a Calf Model of Fibrinonecrotic Bronchopneumonia Induced by Experimental Infection with Chlamydia psittaci
Source: Int J Mol Sci. 2020 Apr 17;21(8):2817. doi: 10.3390/ijms21082817 (PMC7215337; doi:10.3390/ijms21082817)
Supplement: Supplementary file 1 [file ijms-21-02817-s001.pdf]

Supplement Table S1: Absolute data of rectal temperature, respiratory rate, and tidal volume in calves challenged with *Chlamydia psittaci*

Time point	Rectal temperature at baseline[1] (°C)	Rectal temperature at day of necropsy (°C)	Respiratory rate at baseline[1,2] (breath/min)	Respiratory rate before necropsy (breath/min)	Tidal volume at baseline[3] (mL/kg b.w.)	Tidal volume before necropsy (mL/kg b.w.)
	median {range}	median {range}	median {range}	median {range}	median {range}	median {range}
2 dpi (*n*=3)	38,7 {38.3 – 39.0}	41.2 {40.4 – 41.4}.	26 {24 - 32}	60 {56 – 100}	9.1 {8.5 – 9.4}	n.a.[4]
3 dpi (*n*=3)	38.6 {38.3 – 39.0}	**40.8** {40.8 – 41.4}	24 {20 - 32}	48 {44 - 60}	9.6 {8.2 – 11.1}	7.0 {6.5 – 9.4}
4 dpi (*n*=3)	39.1 {38.7 – 39.5}	**40.2** {40.1 – 40.2}	31 {22 - 44}	64 {60 - 68}	9.3 {8.0 – 10.9}	6.7 {5.7 – 7.7}
7 dpi (*n*=3)	38.7 {38.0 – 39.1}	38.6 {38.2 – 38.8}	22 {20 - 28}	24 {24 - 32}	9.3 {7.6 – 10.2}	9.1 {7.1 – 9.9}
10 dpi (*n*=3)	38.5 {38.3 – 39.0}	38.2 {38.1 – 40.6}	25 {24 - 32}	24 {24 - 28}	9.0 {8.5 – 11.5}	10.2 {9.7 – 10.9}
14 dpi (*n*=3)	38.4 {38.2 – 38.5}	38.4 {38.4 – 38.5}	25 {20 - 28}	28 {24 - 40}	8.9 {8.8. – 10.9}	10.6 {8.3 – 12.2}
35/37 dpi (*n*=3)	38.6 {38.5 – 38.7}	38.6 {38.2 – 39.5}	32 {26 - 36}	24 {24 - 28}	8.9 {8.1 – 9.7}	9.0 {8.8 – 9.4}

[1]baseline = mean of individual measurements at two different days in the week before challenge.

[2]Respiratory rate was counted in resting animals (in stable).

[3]Tidal volume is expressed in mL per kg body weight (b.w.) because absolute tidal volumes increase continuously in growing animals.

[4]n.a. = not available (calves necropsied at 2dpi were too sick to underwent pulmonary function testing).
